# *Temnothorax pilagens* sp. n. – a new slave-making species of the tribe Formicoxenini from North America (Hymenoptera, Formicidae)

**DOI:** 10.3897/zookeys.368.6423

**Published:** 2014-01-08

**Authors:** Bernhard Seifert, Isabelle Kleeberg, Barbara Feldmeyer, Tobias Pamminger, Evelien Jongepier, Susanne Foitzik

**Affiliations:** 1 Senckenberg Museum for Natural History Goerlitz, Am Museum 1, D - 02826 Goerlitz, Germany; 2 Evolutionary Biology, Department Biology, Johannes Gutenberg University of Mainz, Johannes von Müller Weg 6, D - 55099 Mainz, Germany

**Keywords:** *Temnothorax*, Nearctic region, dulosis, slave-raiding behavior, morphometrics

## Abstract

A new species of the ant genus *Temnothorax* Forel, 1890 – *Temnothorax pilagens* sp. n. is described from eastern North America. *T. pilagens* **sp. n.** is an obligate slave-making ant with two known hosts: *T. longispinosus* (Roger, 1863) and *T. ambiguus* (Emery, 1895). A differential diagnosis against *Temnothorax duloticus* (Wesson, 1937), the other dulotic congener from the Nearctic, is presented and a biological characteristics of the new species is given.

## Introduction

Three slave-making species of the *Temnothorax* genus group (Bolton 2003) of the ant tribe Formicoxenini are known from North America. Each of the three represents an unmistakable combination of phenotypic characters. They all use species of the genus *Temnothorax* Forel, 1890 as hosts and cluster genetically with species of this genus (Beibl et al. 2005). The first species and outgroup to all the others (Beibl et al. 2005), *Protomognathus americanus* (Emery, 1895), is characterized by an elongated, semi-rectangular head capsule with extremely long antennal scobes that fully accommodate the short and flattened scape when it is folded back. These characters are a convergence to the Holarctic genus *Harpagoxenus* that belongs to the distantly related *Leptothorax* genus group. The second one, *Temnothorax duloticus* (Wesson, 1937), shows an acute, frontoventrally directed dent on the postpetiolar sternite, a high petiole with a bulky, truncate node that slopes down to the caudal cylinder with a distinct step. This particular petiolar shape and the reduction of antennal segments to 11 resemble the situation in the subgenus *Mychothorax* Ruzsky, 1904 of the genus *Leptothorax* Mayr, 1855. However, *Temnothorax duloticus* differs from the latter by the absence of a curved transverse carina on the stipes of the maxillae. On the third species, as yet taxonomically undescribed, only little information exists to date (Herbers and Foitzik 2002, Beibl et al. 2005). Its phenotype is an unmistakable combination of an acute, frontoventrally directed dent on postpetiolar sternite, a stout, hump-backed mesosoma, small scape length, a high petiole that is in lateral aspect roughly triangular, a wide petiole and reduced mandibular dentition. Robin Stuart was the first who recognized the new species (Herbers and Foitzik 2002). We follow his proposal to name this slave-making species *Temnothorax pilagens* sp. n. and provide here the formal taxonomic description and differential diagnosis plus a short comparative life history.

## Material

### Type material

Holotype worker labelled “USA: 44.7560°N, 86.0711°W, Michigan: Sleeping Bear National Lakeshore, 180 m, 2013.05.27 – M509” and “Holotype *Temnothorax pilagens* Seifert et al.”; 3 paratype workers from the holotype nest and the same collecting data; 1 paratype gyne labelled “USA: 44.7560°N, 86.0711°W, Michigan: Sleeping Bear National Lakeshore, 180 m, 2013.05.27 – M502”; 4 paratype workers, each on a separate pin, labelled “USA: 44.8435°N, 86.0612°W, Michigan: North Bar Lake Dunes, 185 m, 2013.05.31 – Q534.3”, “USA: 44.8435°N, 86.0612°W, Michigan: North Bar Lake Dunes, 185 m, 2013.05.31 – Q534.1”, “USA: 44.8435°N, 86.0612°W, Michigan: North Bar Lake Dunes, 185 m, 2013.05.31 – Q534.2”, “USA: 44.8435°N, 86.0612°W, Michigan: North Bar Lake Dunes, 185 m, 2013.05.30 – Q520”; 1 paratype gyne labelled “USA: 44.7560°N, 86.0711°W, Michigan: Sleeping Bear National Lakeshore, 180 m, 2013.05.27 – M502”. Different codes after the date sequence refer to different nests. All material is stored in the Senckenberg Museum of Natural History in Goerlitz.

Comparative material of *Temnothorax duloticus* (Wesson, 1937) consisted of five workers and one gyne labelled “USA: 39.9927°N, 83.2575°W, Ohio: Prairie Oaks Metro Park, 270 m, 2013.06.04 – Z698” and three workers labelled “USA: 40.1469°N, 83.0381°W, Ohio: Highbanks Metro Park, Olentangy River, 239 m, 2013.06.07 - G827”.

## Methods

### Recording of morphological data

Twenty morphometric characters currently being used in taxonomy of Palaearctic *Temnothorax* (Seifert 2006, Csösz et al. 2013) were investigated. In bilaterally recorded characters, arithmetic means of both body sides were calculated. All measurements were made on mounted and dried specimens using a pin-holding stage, permitting full rotations around X, Y, and Z axes. A Leica M165C high-performance stereomicroscope equipped with a 2.0 planapochromatic objective (resolution 1050 lines/mm) was used at magnifications of × 120–384. The mean relative measuring error over all magnifications was 0.3%. A Schott KL 1500 cold–light source equipped with two flexible, focally mounted light–cables, providing 30°–inclined light from variable directions, allowed sufficient illumination over the full magnification range and a clear visualization of silhouette lines. A Schott KL 2500 LCD cold–light source in combination with a Leica coaxial polarized–light illuminator provided optimal resolution of tiny structures and microsculpture at highest magnifications. Simultaneous or alternative use of the cold-light sources depending upon the required illumination regime was quickly provided by regulating voltage up and down. A Leica cross-scaled ocular micrometer with 120 graduation marks ranging over 52% of the visual field was used. To avoid the parallax error, its measuring line was constantly kept vertical within the visual field. Measurements of body parts always refer to real cuticular surface and not to the diffuse pubescence surface.

Z-stack photographs were made with a Leica Z6 APO photomicroscope equipped with 2.0 × planapochromatic objective and the automontage software Leica application suite version 3.

CL maximum cephalic length in median line; the head must be carefully tilted to the position with the true maximum. Excavations of occiput and/or clypeus reduce CL.

CS cephalic size; the arithmetic mean of CL and CW, used as a less variable indicator of body size.

CW maximum cephalic width; the maximum is found in *Temnothorax* and *Leptothorax* usually across and including the eyes.

EYE eye-size index: the arithmetic mean of the large (EL) and small diameter (EW) of the elliptic compound eye is divided by CS, i.e. EYE=(EL+EW)/(CL+CW). All structurally visible ommatidia are considered.

FCDV tangens of divergence angle of frontal carinae measured along a 50 µm section from FRS level caudad. A cross-scaled ocular micrometer and full magnification is used.

FRS distance of the frontal carinae immediately caudal of the posterior intersection points between frontal carinae and the lamellae dorsal of the torulus. If these dorsal lamellae do not laterally surpass the frontal carinae, the deepest point of scape corner pits may be taken as reference line. These pits take up the inner corner of scape base when the scape is fully switched caudad and produce a dark triangular shadow in the lateral frontal lobes immediately posterior of the dorsal lamellae of scape joint capsule (fig. 1 in Seifert 2006).

MGr Depth of metanotal groove or depression, measured from the tangent connecting the dorsalmost points of promesonotum and propodeum; here given as per cent ratio of CS.

MH in workers: with mesosoma in lateral view and measured orthogonal to “longitudinal mesosomal axis”, MH is the longest measurable ***section*** line of mesosoma at mesopleural level (not height above all). “Longitudinal mesosomal axis” in lateral view is defined as straight line from the centre of propodeal lobe to the border point between anterior pronotal shield and propleuron. In gynes it is the longest section line directed perpendicular to the straight dorsal profile line of mesosoma (formed by mesonotum and scutellum). The lower reference point is usually lowest part of mesopleuron.

MW maximum mesosoma width (worker); maximum mesosoma width anteriorly of the tegulae (gynes).

ML in workers: mesosoma length from caudalmost point of propodeal lobe to transition point between anterior pronotal slope and anterior propodeal shield (preferentially measured in lateral view; if the transition point is not well defined, use dorsal view and take the centre of the dark-shaded borderline between pronotal slope and pronotal shield as anterior reference point). In gynes: length from caudalmost point of propodeal lobe to the most distant point of steep anterior pronotal face.

PEH maximum petiole height. The straight section of ventral petiolar profile at node level is the reference line perpendicular to which the maximum height of petiole node is measured.

PEL Diagonal petiolar length in lateral view; measured from anterior corner of subpetiolar process to dorsocaudal corner of caudal cylinder.

PEW maximum width of petiole.

PoOc postocular distance. Use a cross-scaled ocular micrometer and adjust the head to the measuring position of CL. Caudal measuring point: median occipital margin; frontal measuring point: median head at the level of the posterior eye margin. Note that many heads are asymmetric and average the left and right postocular distance (fig. 2 in Seifert 2006).

PPW maximum width of postpetiole.

SL maximum straight line scape length excluding the articular condyle as arithmetic mean of both scapes.

SP maximum length of propodeal spines; measured in dorsofrontal view along the long axis of the spine, from spine tip to a line, orthogonal to the long axis, that touches the bottom of the interspinal meniscus (fig. 3 in Seifert 2006). Left and right SP are averaged. This mode of measuring less ambiguous than other methods but results in some spine length in species with reduced spines.

SPBA the smallest distance of the lateral margins of the spines at their base. This should be measured in dorsofrontal view, since the wider parts of the ventral propodeum do not interfere with the measurement in this position. If the lateral margins of spines diverge continuously from the tip to the base, a smallest distance at base is not defined. In this case, SPBA is measured at the level of the bottom of the interspinal meniscus.

SPST distance between the centre of propodeal stigma and spine tip. The stigma centre refers to the midpoint defined by the outer cuticular ring but not to the centre of real stigma opening that may be positioned eccentrically.

SPTI the distance of spine tips in dorsal view; if spine tips are rounded or thick take the centres of spine tips as reference points.

TrScuC density of transverse microsculpture elements in centromedian vertex. Count the transverse elements crossing a median line of ± 120 µm at a central place with most such elements. Unit: number of elements / mm.

## Results

### Temnothorax pilagens sp. n.

http://zoobank.org/816821D0-8B10-4BEE-889D-CA45A80ABFDD

http://species-id.net/wiki/Temnothorax_pilagens

#### Etymology.

The species epithet refers to the slave raiding behaviour of the new ant species (from Latin: pilare, English: to pluck, plunder, pillage).

#### Description and differential diagnosis.

The differential diagnosis is done in relation to the congeneric slave-making species *Temnothorax duloticus*. Measurements and indices in the text of description are the arithmetic means of the whole samples (for full data see Table 1).

**Figure 1.**
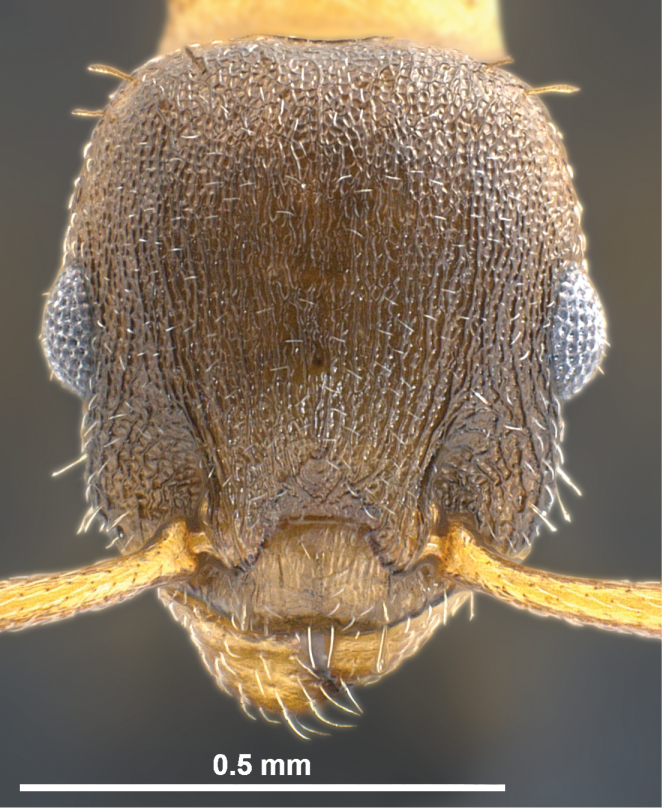
*Temnothorax pilagens* sp. n., worker, head of holotype in dorsal view.

**Worker** (Figs 1, 3 and 5, Table 1): Body size close to the genus average of *Temnothorax*, mean CS 645 µm. Head relatively broader [CL/CW 1.048 but 1.078 in *Temnothorax duloticus*], in dorsal aspect with strongly convex postocular sides and nearly linear, converging genae. Postocular distance smaller [PoOc/CL 0.364 but 0.394 in *Temnothorax duloticus*]. Antennae with 11 segments only, scape strikingly shorter [SL/CS 0.721 but 0.801 in *Temnothorax duloticus*]. Vertex finely longitudinally rugulose, distance between rugulae on central vertex 12 µm. The rugulae are connected by very delicate transverse anastomosae, which have on central vertex a mean distance of 12–14 µm. Clypeus finely longitudinally carinulate and in full-face view with straight or feebly emarginated anteromedian margin. Only the apical and subapical dent of the masticatory margin of the mandibles are fully developed and acute, the following dents are reduced to an undulating line of 3–6 shallow waves [in *Temnothorax duloticus* at least the first three dents are fully developed and the whole dentition is more similar to the normal *Temnothorax* situation]. Genae each with 2–6 semi-erect to erect setae [these are absent in *Temnothorax duloticus*]. Mesosoma massive, in lateral view with strongly convex dorsal profile, appearing hump-backed – i.e., much more compact and shorter than in *Temnothorax duloticus* [ML/CS 1.174 but 1.272 in *Temnothorax duloticus*]. Spines significantly shorter and thicker [SPST/CS 0.364 and SP/CS 0.300 but 0.425 and 0.361 respectively in *Temnothorax duloticus*]; spines in lateral view semi-erect, deviating from longitudinal axis of the mesosoma by 27–35°; in dorsal view diverging by 36–39° and with a larger basal distance [SPBA/CS 0.382, but 0.317 in *Temnothorax duloticus*]. The entire mesosoma exhibits a rugulose-microreticulate sculpture. Petiolar node in lateral view with a straight or weakly concave frontal profile forming with the short dorsal plane an angle of 81–91°; caudal petiolar profile steeply but linearly sloping down to junction with postpetiole [in *Temnothorax duloticus* there is a distinct step in the caudal slope caused by a prolongation of the caudal cylindric part of petiole]. Petiole clearly shorter [PEL/CS 0.452 but 0.517 in *Temnothorax duloticus*]. Postpetiolar sternite in lateral view with a strongly developed, triangular dent, directed anteroventrad, comparable to situation in *Temnothorax duloticus*. Dorsum of petiole node in dorsal view 1.7–2.0 fold wider than long, postpetiole in dorsal view roughly trapezoidal and much wider than in any independent *Temnothorax* species, PPW/CS 0.491. Whole surface of petiolar and postpetiolar nodes coarsely microreticulate. Surface of 1^st^ gaster tergite smooth and shining, but with a very delicate (sculpture lines only 0.5 µm thick), patchily missing microreticulum [in *Temnothorax duloticus* there is nowhere a connected microreticulum – it is reduced to isolated, scattered structures in the form of an “X” or of a matchstick man]. All dorsal body surfaces with setae of medium length. Dorsal head dark brown. Mesosoma, waist and appendages yellowish, propodeum, meso- and metapleuron sometimes darker brownish. Gaster tergites yellowish, often with small brown bands at posterior margin; the first tergite usually shows big brown patches on each side that may fuse medially in some specimens, then covering 70% of total surface.

**Table 1.** Nineteen shape characters and one size character in *Temnothorax pilagens* sp. n. and *Temnothorax duloticus* including data extracted from the photo of a paratype specimen of *Temnothorax duloticus* F- and p-values of an univariate ANOVA are given and the shape variables are arranged by decreasing F.

	*Temnothorax pilagens* (n=8)	ANOVA F p	*Temnothorax duloticus* (n = 6)	*Temnothorax duloticus* photo of paratype
CS<br/> [µm]	645 ± 29<br/> [603, 684]	0.22<br/> n.s.	639 ± 22<br/> [616, 678]	562
PoOc/CL	0.364 ± 0.006<br/> [0.354, 0.371]	91.76<br/> <0.001	0.394 ± 0.005<br/> [0.388, 0.400]	0.377
SL/CS	0.721 ± 0.019<br/> [0.684, 0.742]	87.55<br/> <0.001	0.801 ± 0.008<br/> [0.785, 0.807]	0.831
ML/CS	1.174 ± 0.027<br/> [1.142, 1.229]	55.96<br/> <0.001	1.272 ± 0.020<br/> [1.242, 1.300]	1.296
PEL/CS	0.452 ± 0.011<br/> [0.438, 0.466]	50.18<br/> <0.001	0.517 ± 0.023<br/> [0.487, 0.538]	0.538
SPST/CS	0.368 ± 0.015<br/> [0.343, 0.383]	43.71<br/> <0.001	0.425 ± 0.017<br/> [0.397, 0.440]	no data
SP/CS	0.300 ± 0.014<br/> [0.268, 0.311]	41.90<br/> <0.001	0.361 ± 0.022<br/> [0.327, 0.392]	no data
SPBA/CS	0.382 ± 0.027<br/> [0.351, 0.411]	21.23<br/> 0.001	0.317 ± 0.021<br/> [0.296, 0.354]	0.281
MPGR/CS [%]	0.13 ± 0.27<br/> [0.0,0.8]	14.60<br/> 0.002	0.89 ± 0.47<br/> [0.1,1.4]	1.09
PnHL/CS	0.158 ± 0.008<br/> [0.146, 0.172]	11.86<br/> 0.005	0.179 ± 0.014<br/> [0.164, 0.197]	no data
PEH/CS	0.425 ± 0.014<br/> [0.400, 0.444]	9.00<br/> 0.011	0.451 ± 0.019<br/> [0.425, 0.476]	0.440
CL/CW	1.048 ± 0.023<br/> [1.019, 1.084]	7.00<br/> 0.021	1.077 ± 0.014<br/> [1.058, 1.099]	1.076
MW/CS	0.656 ± 0.014<br/> [0.635, 0.672]	2.77<br/> n.s.	0.644 ± 0.014<br/> [0.630, 0.671]	0.630
PPW/CS	0.491 ± 0.018<br/> [0.452, 0.506]	2.06<br/> n.s.	0.503 ± 0.012<br/> [0.489, 0.520]	0.468
TrScuC [n / mm]	77.2 ± 5.4<br/> [69,85]	2.02<br/> n.s.	73.5 ± 4.0<br/> [71,80]	70
EYE/CS	0.236 ± 0.008<br/> [0.229, 0.249]	1.73<br/> n.s.	0.231 ± 0.005<br/> [0.225, 0.237]	no data
PEW/CS	0.297 ± 0.010<br/> [0.284, 0.313]	1.34<br/> n.s.	0.304 ± 0.012<br/> [0.284, 0.318]	0.306
SPTI/CS	0.469 ± 0.026<br/> [0.434, 0.505]	0.51<br/> n.s.	0.479 ± 0.030<br/> [0.445, 0.527]	no data
FRS/CS	0.389 ± 0.007<br/> [0.378, 0.399]	0.10<br/> n.s.	0.390 ± 0.010<br/> [0.376, 0.404]	0.381
MH/CS	0.569 ± 0.018<br/> [0.550, 0.600]	0.10<br/> n.s.	0.572 ± 0.026<br/> [0.534, 0.604]	0.577

**Gyne** (only one gyne was evaluated in both *Temnothorax pilagens* sp. n. and *Temnothorax duloticus*): Head size similar to the genus average of *Temnothorax*, mean CS 673 µm. Head very short [CL/CW 0.982 but 1.044 in *Temnothorax duloticus*], in full-face view with strongly convex postocular sides, a feebly concave occipital margin and linear, converging genae. Postocular distance very short [PoOc/CL 0.341 but 0.401 in *Temnothorax duloticus*]. Antennae with 11 segments only, scape very short [SL/CS 0.683 but 0.748 in *Temnothorax duloticus*]. Vertex longitudinally rugulose, distance between rugulae on central vertex 15 µm, the interspaces between rugulae with reticulate microsculpture. Clypeus finely longitudinally carinulate and in full-face view with feebly notched anteromedian margin. The three apical dents of the mandibular masticatory margin are fully developed and acute, the following four dents are reduced to denticles. Mesomoma very small for *Temnothorax* in general, but not smaller than in *Temnothorax duloticus* [ML/CS 1.484, MW/CS 0.904, MH/CS 0.868]. Spines well-developed and acute but significantly shorter and relatively thicker than in *Temnothorax duloticus* [SPST/CS 0.364 and SP/CS 0.265 but 0.457 and 0.360 respectively in *Temnothorax duloticus*]; spines in lateral view very weakly erected, deviating from longitudinal axis of mesosoma by 20°; in dorsal view with a very large basal distance and weakly diverging [SPBA/CS 0.482, SPTI/CS 0.479; in *Temnothorax duloticus* more clearly diverging, SPBA/CS 0.434 and SPTI/CS 0.530]. Whole mesosoma with rugose-microreticulate sculpture that is on mesonotum and mesopleuron less developed. Petiolar node in lateral view very high and with a weakly concave frontal profile forming with the short dorsal plane an angle of 80°; caudal petiolar profile steeply and almost linearly sloping down to junction with postpetiole [in *Temnothorax duloticus* there is a distinct step in the caudal slope caused by a significant prolongation of the caudal cylindric part of petiole]. Petiole clearly shorter than in *Temnothorax duloticus* [PEL/CS 0.496 vs. 0.551]. Postpetiolar sternite in lateral view with a strongly developed, triangular dent, directed anteroventrad, comparable to situation in *Temnothorax duloticus*. Dorsum of petiole node in dorsal view 1.9fold wider than long, postpetiole in dorsal view broadly cordate and much wider than in any independent *Temnothorax* species, PPW/CS 0.491. Whole surface of petiolar and postpetiolar nodes strongly microreticulate. Surface of 1^st^ gaster tergite smooth and shining but with a very delicate (sculpture lines only 0.5 µm thick), patchily missing microreticulum [in *Temnothorax duloticus* the microreticulum more incomplete – frequently reduced to isolated, scattered structures in the form of an “X” or of a matchstick man]. All dorsal body surfaces with setae of medium length, the longest on occiput are 74 µm long. Head, mesosoma and waist brown, appendages yellowish to yellowish brown. Gaster tergites yellowish brown, a lighter yellowish patch is at the base of 1^st^ tergite.

## Discussion

The original description of *Temnothorax duloticus* and the photos in antweb.org (CASENT0103163) of a paratype specimen of the type colony from Ohio: Jackson Country: White’s Gulch clearly show the heterospecifity of *Temnothorax pilagens*. The conspecifity of our two *Temnothorax duloticus* samples with the paratype is indicated by agreement in mesosomal and petiolar shape characters and by the similarity of NUMOBAT data. Both spines of the paratype are broken off – excluding to assess the characters SP, SPTI and SPST. Furthermore, the spatial adjustment of the photo excluded estimating the characters EYE and PnHL. The remaining 15 characters (see Tab. 1) could be extracted from the image – albeit with some distortion. Using these characters and running the paratype in an LDA as wild-card, it is allocated with a posterior probability of p = 1.000 to the same cluster with our *Temnothorax duloticus* samples. The same clear allocation is provided by the 1^st^ factor of a principal component analysis being -0.87 ± 0.20 [-1.07, -0.42] in eight specimens of *Temnothorax pilagens* sp. n. and 0.94 ± 0.37 [0.34, 1.28] in seven specimens of *Temnothorax duloticus*, with the paratype scoring 1.28.

**Figure 2.**
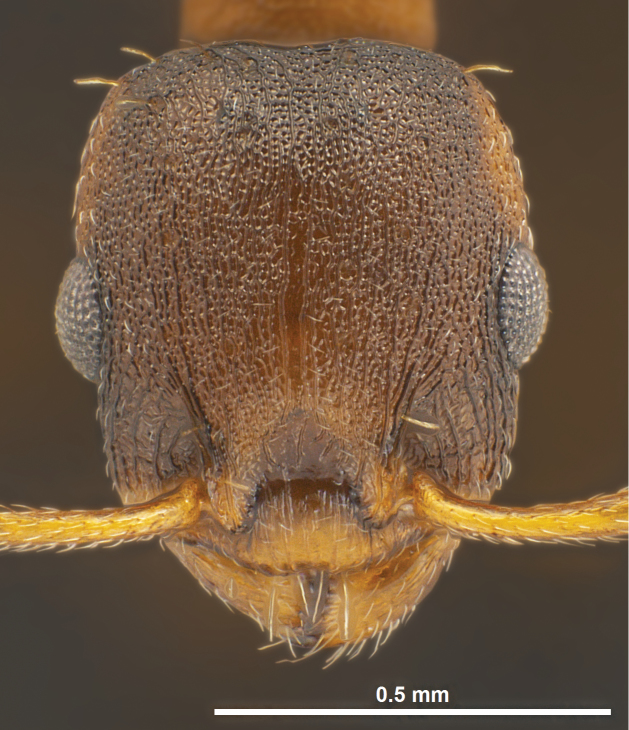
*Temnothorax duloticus*, worker, head in dorsal view.

**Figure 3.**
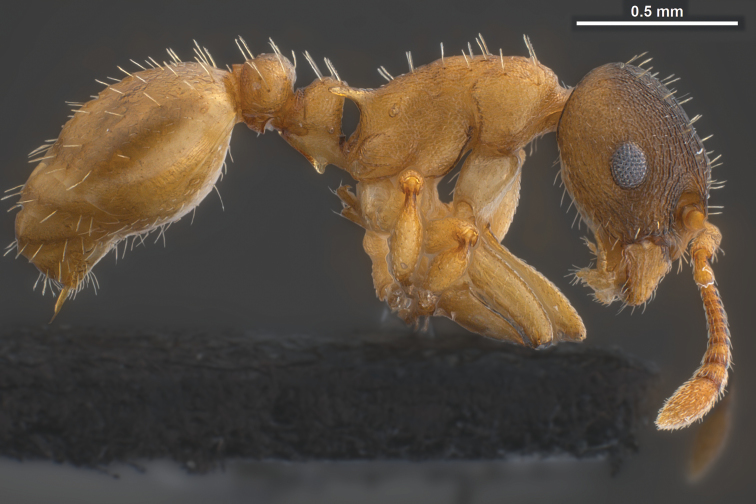
*Temnothorax pilagens* sp. n., worker, holotype in lateral view.

**Figure 4.**
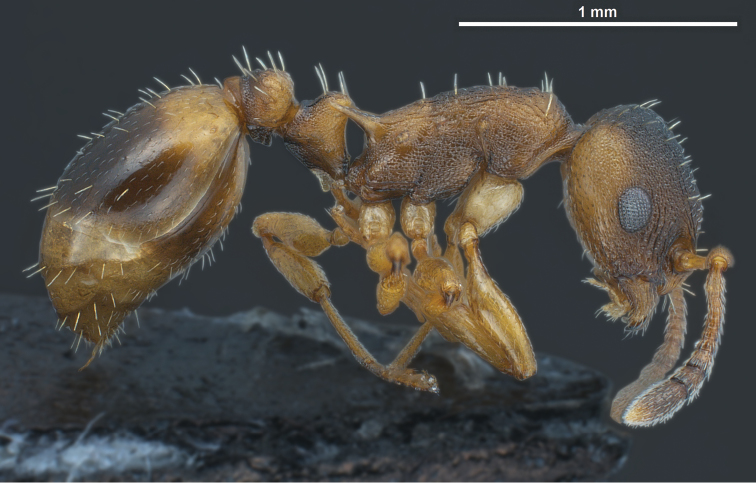
*Temnothorax duloticus*, worker, lateral view.

**Figure 5.**
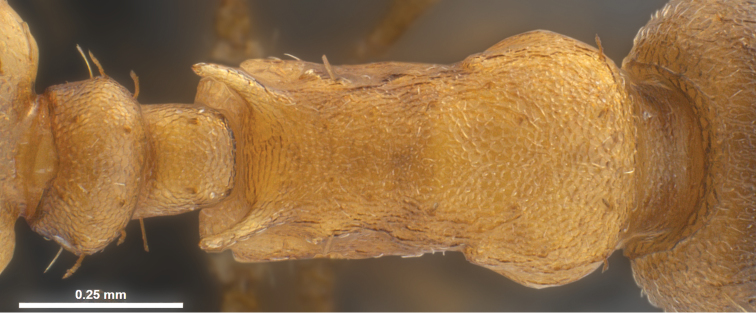
*Temnothorax pilagens* sp. n., worker, mesosoma of holotype in dorsal view.

**Figure 6.**
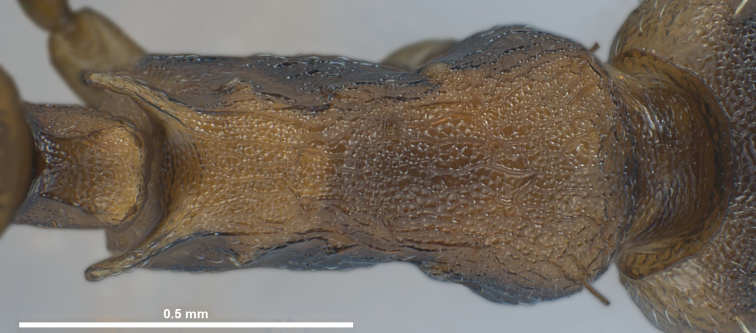
*Temnothorax duloticus*, worker, mesosoma in dorsal view.

Several discriminatory characters allow easy separation of *Temnothorax pilagens* sp. n. and *Temnothorax duloticus* workers. Despite low sample size, there are highly significant differences in 55% of the tested characters (Tab. 1). The characters SL/CS or PoOc/CL alone should provide a safe and parsimonious numeric species delimitation. There is also no doubt that experienced observers can distinguish the two dulotic species by simple eye-inspection integrating subjective impressions on mesosomal, petiolar, cephalic and spine shape and mandibular dentition (compare Figs 1–6).

### Short biological characteristics of *Temnothorax pilagens* sp. n.

**Biology and host species.** Obligate slave-making ant with two known hosts: *Temnothorax longispinosus* (Roger, 1863) and *Temnothorax ambiguus* (Emery, 1895). Mitochondrial DNA phylogeny indicates sister species relationship with *Temnothorax longispinosus* (Beibl et al. 2005). **Geographical range.** Nearctic. North-eastern parts of the United States and possibly south-eastern Canada. **Habitat.** Forest, woodland, parks. Preferentially wooded sites with little understory, and a high density of suitable nest sites, such as acorns, hickory nuts and sticks. **Abundance.** Patchy – occurrence depends on high density of suitable host populations; so far only known from three sites in the Northern US: Niquette Bay State Park, Vermont (44.3513°N, 73.1156°W; 8 colonies collected in 1986; Herbers and Foitzik 2002), E.N. Huyck Preserve, Rensselaerville, New York (42.3133°N, 74.1012°W; 7 colonies collected in 2002 and 2003; Beibl et al. 2005) and Sleeping Bear National Lakeshore, Empire, Michigan (44.7560°N, 86.0711°W, 6 and 44 colonies collected in 2011 and 2013, respectively). In all three populations, *Temnothorax pilagens* was enslaving *Temnothorax longispinosus* and *Temnothorax ambiguus*; many nests with slaves of both host species. In Vermont and New York, this species has not been re-collected recently, despite regular search by our group. Plotting data from 2011 in Michigan: between 0.08 and 0.02 slave-making colonies per m^2^ (at an average of 4.66 host colonies per m^2^). *Temnothorax pilagens* occurs more often in sites with both host species, than in areas with *Temnothorax longispinosus* colonies only (Fisher Test; p = 0.019). We did not sample *Temnothorax* communities with *Temnothorax ambiguus* only. *Temnothorax pilagens* colonies more often contained a mixed slave workforce than *s* laves of a single host species (Chi_1_^2^ = 49.59, p < 0.001). **Nest construction.** As its hosts, *Temnothorax pilagens* nests occur in preformed cavities in acorns, hickory nuts or sticks. **Colony demography.** Strictly monogynous. Most likely polydomous at least during the summer season: 72% of the nests were queenless with queenright nests close-by and neighboring nests merged in the laboratory without aggression. Nests contain on average four slave-making workers (ranging from 0 to 16 *Temnothorax pilagens* workers) and 13 *Temnothorax* slaves (ranging from 2 to 50 workers) – but see Herbers and Foitzik (2002) for a nest with 27 *Temnothorax pilagens* and 55 slave workers. **Colony foundation.** Four colonies with no *Temnothorax pilagens* workers, but a founding queen were collected. All four contained *Temnothorax longispinosus* slaves only. **Slave-raids.** Obligatory slave hunter of *Temnothorax longispinosus* and *Temnothorax ambiguus*. Raids are performed either by a scout alone or via group recruitment of up to four slavemaking workers forming a raiding column headed by a scout. Slave raids resemble more those of its congener *Temnothorax duloticus* than that of *Protomognathus americanus* (Alloway 1979). This is especially true for the frequent and effective use of the stinger in fights: well-aimed stings from a caudal direction between head and thorax cause paralysis in hosts followed by quick death. Effective sting use is likely facilitated by morphological adaptations, such as strongly developed flexor-muscles in the petiole and postpetiole allowing for easy gaster flexion. Similar behavioural strategies and morphological traits are also found in *Temnothorax duloticus*, but not in *Protomognathus americanus*. Hosts attacked by *Temnothorax pilagens* show little or only delayed flight responses. Occasionally, host workers try to drag slavemakers out of the nest, and only respond aggressively when attacked by them. The low host responsiveness towards invading *Temnothorax pilagens* indicate reduced or supressed enemy recognition. Due to their most effective stinging behaviour, *Temnothorax pilagens* can cause high rates of host casualties (ranging from 5% to 100%). As the high variation in the rate of casualties indicates, raids can be highly aggressive or relatively peaceful; the latter was often found in raids against queenless host nests. Slave-makers do not only take brood from the attacked host nests, but in 6 of the observed 11 raids, they also carry adult host workers back to their nest and integrated them into the slave workforce.

## Supplementary Material

XML Treatment for Temnothorax pilagens
